# Reflections on a Research Clerkship Abroad

**DOI:** 10.1007/s13187-015-0874-4

**Published:** 2015-07-11

**Authors:** Bertha Eisses, Jakob de Vries, Shine Chang

**Affiliations:** The University Medical Center Groningen, Hanzeplein 1, PO Box 30.001, 9700 RB Groningen, The Netherlands; Cancer Prevention Research Training Program, Division of Cancer Prevention and Population Sciences, The University of Texas MD Anderson Cancer Center, Houston, TX USA

## Student Perspective

Medical students at the UMCG are introduced to the basic principles of medical research during the 3-year bachelor’s period. These experiences increase awareness of the importance of research and give them the competency to explore the literature and to find the gaps and limits of health science. In the 3-year master’s period, students practice these skills during a research clerkship of 26 weeks. Many opportunities are offered locally, but students can also choose to go abroad.

Personally, I wanted to go to a famous oncology research center in the world, because I want to pursue a career in oncology. I was lucky to find a supervisor who knew about The University of Texas MD Anderson Cancer Center, and I was immediately enthusiastic. A great advantage to Texas was having a family member living close to Houston. In March 2014, I had the first encounter with my supervisor at UMCG and soon after that I contacted my supervisor in Houston. In June, I started writing my proposal and organizing the required documents and could start my elective in November.

Planning a research project abroad is not easy. You need sponsorship letters from your university and from your sponsoring supervisor abroad for obtaining a visa from the consulate. I had to create an online profile on the MD Anderson website for uploading the required documents. And besides that, you need letters to apply for funding.

Every student has to select a research topic, review literature, generate data, and interpret research findings. However, in doing a research project abroad, you have to deal with a lot more. For example, you need to find a place to stay short-term, to learn about other cultures, and sometimes to speak a foreign language, which is not always easy, especially in presenting research. Also, you need to navigate public transport or buy a car. I decided to travel by bicycle, which was uncommon in Houston. Finally, if you have problems, your friends and family are not easily available to you for support.

I experienced many benefits from my international research experience at MD Anderson: attending lectures given by faculty from a top international institution and invited scientific leaders from elsewhere, being exposed to research methods from an international context, practicing how to work in a foreign language, improving my social skills, expanding my cultural awareness, growing my professional network, and learning how to cope with unexpected situations (e.g., organizing visa documents). I learned how cancer registries work, how their limitations and benefits influence the research questions asked, how to analyze data, including how to manage outliers and small numbers, and how to analyze and present research outcomes. Of course, adding this unique opportunity to my CV was a highlight.

I was fortunate to have ideal supervisors. They were available anytime, including after 5 p.m., which helps when time is short. They understood and respected my position as a student and knew the extent of their responsibilities to help me with my clerkship research project. Having knowledgeable supervisors is critical to a successful traineeship, in that they have experience managing research projects and trainee appointments or know people who can help.

Thus, in my experience, a successful research experience abroad requires skills in organization, time management, good communication, and collaboration. One must also have flexibility, perseverance, focus, ability to work from priorities, and openness to learning in order to take full advantage of the many opportunities available in a research clerkship abroad. The ability to develop these skills early in your career will help to accomplish future goals and long-term career success.

## Sponsoring a Medical Student for an International Research Clerkship

William Glasser told us in 1978 that learning by teaching is far more effective than learning by lecturing. In Groningen at UMCG, we think that learning by (re)searching could be even more effective. In fact, this is the basis of problem-based learning. Students learn how to learn in the first years, building the foundation of their body of knowledge and acquiring academic skills that result in a bachelor thesis in their third year. During the master’s phase, the whole process of designing a research question, writing a proposal, performing the research, and analyzing and presenting the results should be practiced in the research clerkship. Two key competencies are most relevant: (1) critical evaluation of information and its sources and application of this knowledge appropriately to practice decisions and (2) contribution to the creation, dissemination, application, and translation of new medical knowledge and practices.

I was fortunate to connect my student, Bertha, to my colleague, Shine Chang, Ph.D., at MD Anderson. Dr. Chang and I share an interest in cancer education with many others in both the American and European Associations for Cancer Education (AACE and EACE, respectively). During the preparation of this clerkship, we had discussions about the topic and anticipated some formal hurdles to come. This was part of the learning process and tested our perseverance. Good collaboration and communication between the three of us and the educational office(r)s were essential to get approval at all levels, at both institutions.

The organizational hurdles sometimes felt more challenging than the content of the clerkship project being proposed! However, MD Anderson proved to be an excellent place for Bertha to work on her academic skills, receiving full support and coaching even when a change in the topic was necessary. In that situation, I was able to find a new content expert at UMCG to support Bertha in this stressful situation. In spite of this minor setback, her project was completed very successfully with a high grade on Bertha’s master thesis, a prize winning presentation of this process at the EACE meeting in Heidelberg, a first draft of a publication for a scientific journal, and last but not least, professional and personal growth of young medical professional.

## Hosting an International Medical Student for a Research Clerkship

In hosting an international medical student for a research clerkship, I found four phases to the process: preparing for the clerkship, starting the experience, doing the work, and finishing the experience. Preparing for the clerkship involves two kinds of paperwork: first, the hosting faculty must complete all the legal and administrative processing for the research experience. Although every institution will have its unique requirements, getting through the process to appoint an international student can take considerable time, often longer than you might expect; start early. The second preparatory paperwork involved the team in a collaborative creation of a research and educational proposal for approval by Bertha’s medical school that described the planned experience for the research clerkship. To get approval for the proposal from the school before starting the clerkship requires the student to assimilate a lot of information quickly, which can be facilitated with examples of proposals, frequent videoconferencing, and trading drafts with feedback. Ideally, designing the project as a publishable manuscript or at least as an abstract that can be submitted for presentation at a scientific meeting is desirable, but not required.

In the second phase, the hosting supervisor needs to be ready to help the student get oriented and into the research project quickly. One suggestion is to use resources available at your research medical library, including tutorials on citation database searching, citation software, and other online resources that are helpful to researchers. To enrich her experience, I encouraged her to attend as many seminars as were interesting to her, to take tours of the institution, as well as other nearby facilities, and to meet and spend time with other trainees. She shadowed clinical faculty, conducted informational interviews [1], and participated in educational activities for trainees. These activities helped her practice and strengthen her networking skills, which are particularly useful when having a professional experience far from home. For example, we had her give a presentation about cancer trends and prevention activities in her home country early in her clerkship. Such an assignment helped her practice presentation skills as a small “stretch” assignment—finding, learning and organizing information, designing slides, practicing the presentation—on a topic that she is comfortable with—her own country. This first presentation allowed me to gauge her presentation skills early in her clerkship, which helped me know how much preparation she would need for presenting her research near the end of her clerkship.

The third phase centers on finishing the research project. As noted, unanticipated problems occur, so having alternate plans is helpful. As a hosting supervisor, having a variety of data resources available, as well as feasible research questions, is advantageous. Once the research project is launched, careful attention to a timeline with milestones is critical for getting the written report finished because the clerkship is not only brief, but goes by quickly. For this reason, writing should be started and the organization of the presentation be scheduled as early as possible, as should the celebration before the student leaves to go home. Because a medical student will have had initial exposure to research methods and statistics, I highly recommend that statistical and programming support be available to allow the student to focus on planning the research and interpreting results, not struggling to derive the results. Meetings need to be scheduled regularly as the learning stages may be accelerated, requiring more frequent discussions, albeit sometimes brief (e.g., to confirm interpretations, to decide analytic strategies).

The final phase focuses on ensuring the successful completion of the research clerkship. Time must be set aside for practicing the presentation, as well as multiple iterations of the written document. In Bertha’s case, she was required to write in both Dutch and English, which required switching between subtly different scientific formats and writing styles. As Bertha was already preparing to present her research to her colleagues at MD Anderson, it was easy to develop an abstract from the research and presentation, and given the timing of the EACE meeting, to create an educational abstract as well. Finally, not every task can be completed before the clerkship ends, so having a plan to finish components after the student leaves is important for successful completion of unfinished work. Such a plan can include specific deadlines selected with the activities and projects that she has scheduled for her return, as well as alternate plans to ensure the completion of the research.
